# Artificial Intelligence in Medicine: A Multinational Multi-Center Survey on the Medical and Dental Students' Perception

**DOI:** 10.3389/fpubh.2021.795284

**Published:** 2021-12-24

**Authors:** Sotirios Bisdas, Constantin-Cristian Topriceanu, Zosia Zakrzewska, Alexandra-Valentina Irimia, Loizos Shakallis, Jithu Subhash, Maria-Madalina Casapu, Jose Leon-Rojas, Daniel Pinto dos Santos, Dilys Miriam Andrews, Claudia Zeicu, Ahmad Mohammad Bouhuwaish, Avinindita Nura Lestari, Lua'i Abu-Ismail, Arsal Subbah Sadiq, Almu'atasim Khamees, Khaled M. G. Mohammed, Estelle Williams, Aya Ibrahim Omran, Dima Y. Abu Ismail, Esraa Hasan Ebrahim

**Affiliations:** ^1^Department of Neuroradiology, The National Hospital for Neurology and Neurosurgery, University College London NHS Foundation Trust, London, United Kingdom; ^2^Department of Brain Repair and Rehabilitation, Queen Square Institute of Neurology, University College London, London, United Kingdom; ^3^University College London Medical School, University College London, London, United Kingdom; ^4^Computer Science Department, University College London, London, United Kingdom; ^5^School of Medicine, Nottingham University, Nottingham, United Kingdom; ^6^Faculty of Dental Medicine, Carol Davila University of Medicine and Pharmacy, Bucharest, Romania; ^7^NeurALL Research Group, School of Medicine, Ecuador Universidad Internacional del Ecuador, International University of Ecuador, Quito, Ecuador; ^8^Department of Radiology, University Hospital of Cologne, Cologne, Germany; ^9^School of Medicine, Cardiff University, Cardiff, United Kingdom; ^10^Department of Clinical Neurophysiology, The National Hospital for Neurology and Neurosurgery, University College London NHS Foundation Trust, London, United Kingdom; ^11^Faculty of Medicine, University of Tobruk, Tripoli, Libya; ^12^School of Medicine, Universitas Islam Bandung, Bandung, Indonesia; ^13^School of Medicine, Yarmouk University, Irbid, Jordan; ^14^CMH Medical College Lahore, Lahore, Pakistan; ^15^School of Medicine, Tanta University, Tanta, Egypt; ^16^Peninsula Dental School, University of Plymouth, Plymouth, United Kingdom; ^17^School of Medicine, Hashemite University, Zarqua, Jordan; ^18^School of Medicine, Sabha University, Sabha, Libya

**Keywords:** artificial intelligence, dental students, medical students, medicine, survey

## Abstract

**Background:** The emerging field of artificial intelligence (AI) will probably affect the practice for the next generation of doctors. However, the students' views on AI have not been largely investigated.

**Methods:** An anonymous electronic survey on AI was designed for medical and dental students to explore: (1) sources of information about AI, (2) AI applications and concerns, (3) AI status as a topic in medicine, and (4) students' feelings and attitudes. The questionnaire was advertised on social media platforms in 2020. Security measures were employed to prevent fraudulent responses. Mann-Whitney *U*-test was employed for all comparisons. A sensitivity analysis was also performed by binarizing responses to express disagreement and agreement using the Chi-squared test.

**Results:** Three thousand one hundred thirty-three respondents from 63 countries from all continents were included. Most respondents reported having at least a moderate understanding of the technologies underpinning AI and of their current application, with higher agreement associated with being male (*p* < 0.0001), tech-savvy (*p* < 0.0001), pre-clinical student (*p* < 0.006), and from a developed country (*p* < 0.04). Students perceive AI as a partner rather than a competitor (72.2%) with a higher agreement for medical students (*p* = 0.002). The belief that AI will revolutionize medicine and dentistry (83.9%) with greater agreement for students from a developed country (*p* = 0.0004) was noted. Most students agree that the AI developments will make medicine and dentistry more exciting (69.9%), that AI shall be part of the medical training (85.6%) and they are eager to incorporate AI in their future practice (99%).

**Conclusion:** Currently, AI is a hot topic in medicine and dentistry. Students have a basic understanding of AI principles, a positive attitude toward AI and would like to have it incorporated into their training.

## Introduction

In general, artificial intelligence (AI) refers to the concept of automated machines able to perform human tasks (1). AI is a fast-paced developing field, with many applications already being available for use in our daily life activities (e.g., speech-/text-recognition, email spam-filters) (2) and emerging uses attracting a great deal of attention in Medicine and Dentistry over the past decade (3). It has already started to impact on specialties such as radiology (4), pathology (5), dermatology (6), and dentistry (7) especially in the developed world. However, it has been postulated to revolutionize global health in low- and middle-income countries as well because of its ability to apply novel analytical methods in large datasets related to complex diagnostic tasks (8). Throughout the world, the advent of commercially available AI products are heavily advertised and discussed in the medical field as specialized robots and algorithms have the potential to dramatically assist and possibly replace the human physician (9). As AI is yet to be fully established in Medicine and Dentistry, it will have the biggest impact on the next generations of doctors and dentists, respectively. Recently, a small cohort study in Germany found out that medical students have a positive attitude toward integrating AI in the medical procedures (10). However, it remains uncertain whether medical and dental students in general are concerned that AI could significantly affect the current practice of clinical and academic medicine in the future.

A conceptual framework (11) discussing the prospect of incorporating AI in the medical training has been recently designed (12). Although the framework discusses AI content to be included in the curriculum and administrative issues which may need to be overcome, the desire of the medical and dental students to be taught AI related concepts is yet to be elucidated. Although similar surveys have been performed (10, 13, 14), the topic remains relevant because (1) the hype around AI is unprecedented, (2) the impact of AI in Medicine is evolving as currently there is no AI widely adopted and infallible, and (3) the perception of stakeholders may change over time even in the short term. In addition, to the best of our knowledge, our survey is the first one to explore this topic from a multinational perspective. Thus, we designed an electronic survey to assess attitudes of undergraduate students in Medicine and Dentistry toward AI. We chose to include both medical and dental students as: (1) their training is similar (i.e., divided in pre-clinical and clinical years) based on a foundation of biology and chemistry disciplines (i.e., physiology, biochemistry etc.) and (2) AI is a fast-growing field in both Medicine and Dentistry.

## Methods

### Ethical Considerations

According to the University College London Research Ethics Committee guidelines, ethical approval was not required for our study and a waiver from the Joint Research Office (University College London/University College London Hospitals) was applied (15). All procedures performed were in accordance with the ethical standards of the institutional and/or national research committee and with the 1964 Helsinki declaration and its later amendments or comparable ethical standards. Participation was completely voluntary and data collection was entirely anonymous by design. All the respondents provided informed consent after being instructed on the nature and purpose of the survey and were offered the possibility to withdraw at any time.

### Study Design

An electronic survey was designed using Google Forms (Google LLC, United States). The survey was divided into five subsections. The first subsection aimed to gather general demographics data including: age, gender, student category (medical/dental), year of study, and self-reported tech-savviness. Tech-savviness was scored using a four-point Likert scale (strongly disagree, somewhat disagree, somewhat agree, strongly agree). The second subsection explored the understanding on AI basic principles (e.g., machine learning), daily life use (e.g., speech-/text-recognition, email spam-filters) and personal sources of information (e.g., media, social medical, university, friends/family, web browsing). The third subsection aimed to explore the understanding of AI as a topic in Medicine and Dentistry asking whether the participant is aware that: (1) AI in medical research attracted more investment than any AI projects in any other fields, and (2) AI and deep learning are broadly discussed in the medical community. The fourth subsection assessed students' feelings and attitudes toward AI exploring whether the respondent: (1) perceives AI as a partner or a competitor, (2) believes that physicians will be replaced in the foreseeable future, (3) is frightened or excited by the developments, and (4) thinks that AI will improve medicine and would like it to be incorporated into the medical/dental training. These sections were scored via three types of five-point Likert scales: (1) not at all, to a little extent, to a moderate extent, to a great extent and to a very great extent, (2) not at all aware, not so aware, somewhat aware, very aware, extremely aware, and (3) strongly disagree, somewhat disagree, undecided, somewhat agree, strongly agree. The fifth subsection consisted of two short-answer questions related to what the respondent finds exciting about AI in the medical field, and what they would like to hear/learn about AI during their medical/dental studies.

### Recruitment

Facebook has emerged as a tool for survey data collection given its targeted advertisements (16). Thus, the questionnaire was advertised on Facebook to undergraduate medical and dental students across the world. As such, we used a generic Facebook advertising campaign to reach users who specify that they are currently a student of Medicine or Dentistry. To avoid restricting the target population, we did not specify any other characteristics for 1 month. Then, we have evaluated the demographic characteristics of the participants and noticed that females, those from a developing country, dental students, and clinical students were under-represented. Thus, we created four additional advertising campaigns for another month to target underrepresented groups aiming for: 55–70% females, 20–30% from a developed country, 10–30% dental students, and 50% clinical students. We did not try to enforce stricter ratios because: (1) it is unclear what the ratios are globally, (2) there is geographical variation in the ratios, and (3) to avoid attrition bias as the Facebook targeting algorithm is more likely to show the advertisement to individuals having similar characteristics to the ones that have been previously interacted with the ad.

During the Facebook advertising period, the survey had a distributor recruitment rubric at the end at the end of the questionnaire. From the 173 students who have shown interest, only 45 agreed to comply with the distribution requirements and actively participated in the process. In an attempt to obtain an unbiased sample, we have asked the distributors to: (1) write to their medical/dental school and inquire whether they would be willing to distribute the survey to the whole cohort; (2) attempt to gain access to social media groups (Facebook, What's App etc.) containing all students from each year of study; (3) don't send the survey to their friends or to any groups not containing all the year students and (4) translate the study into their language if necessary, appropriate or feasible.

To prevent fraudulent responses a series of security measures were implemented (17): (1) the responses were limited to one only; (2) Completely Automated Public Turing test to tell Computers and Humans Apart (CAPTCHA) script was created and implemented into the Google Form; (3) students were required to specify their medical school; (4) two short-answer questions were included and awards advertised for the best answers; and (5) collected data was checked for inconsistencies. The data collection period was between 11/04/2020 and 01/10/2020.

### Statistical Analyses

After the closing date, the results were downloaded. Tech-savviness was binarized as follows: 0 = strongly or somewhat disagree and 1 = somewhat or strongly agree. For each other question, the categories were preserved as above and recoded numerically from 0 to 4. The variance of its question was also recorded.

Comparison of the distribution of responses for each second, third, and fourth section questions were evaluated for the following categories: (1) male vs. female, (2) tech-savvy vs. non-tech-savvy, (3) pre-clinical vs. clinical, (4) dental vs. medical student, (5) young (aged 21 or younger) vs. mature (aged 22 or older) as defined by the UK's University and Colleges Admissions Services (UCAS) (18), and (6) from a developed vs. from a developing country as defined by the United Nations (19). In addition, we have analyzed the distribution of responses for the second, third, and fourth sections per country for any state having more than 50 respondents.

Distribution of data was assessed visually on histograms. Given the non-normal distribution of categorical data, Mann-Whitney *U*-test was employed for all comparisons.

A sensitivity analysis was also performed by binarizing responses to express disagreement (strongly or somewhat disagree, not at all, to a little extent, not at all or not so aware) and agreement (somewhat or agree, to a moderate or great extent or very great extent, and somewhat or very or extremely aware). Pearson's Chi-squared test with Yates' continuity correction was employed for all comparisons.

As most of our respondents regarded themselves as tech-savvy, we performed an additional sensitivity analysis. We considered the Likert response scale an ordinal scale and used generalized linear models with ordinal logit link (i.e., ordinal logistic regression) to assess whether the above associations persisted after adjusting for tech-savviness. The proportional odds assumption for ordinal logistic regression was tested using a Brant test (20).

A *p* < 0.05 was considered statistically significant. All analyses were performed using R version 3.6.3.

## Results

Participant characteristics are summarized in Table 1, while full survey results are presented in Table 2. A breakdown of respondents per country is presented in Supplementary Table 1. The participants were more likely be female (66.5%) and study pre-clinical (55.8%) medicine (79.6%) in a developing country (73.6%). In addition, the slight majority of the participants were aged 22 or older (51.7%) with a mean age of 22.0 ± 2.8 years, and mostly self-rated themselves as being tech-savvy (79.5%).

**Table 1:** Participant demographics, sources of information on artificial intelligence (AI), perceived potential applications of AI in medicine/dentistry, and concerns on AI.

**Participant characteristics (*n* = 3,133)**	***n*** **= 3,133**
Age, years	21.95 ± 2.77
Males, (%)	1,050 (33.51)
Tech-savvy^a^, (%)	2,489 (79.45)
Medical students, (%)	2,495 (79.63)
Clinical students, (%)	1,385 (44.21)
From a developed country^b^, (%)	828 (26.43)
**Sources of information on artificial intelligence**	
Media, (%)	1,265 (40.38)
Social media, (%)	1,861 (59.40)
Web browsing, (%)	1,883 (60.10)
Friends or family, (%)	772 (24.64)
University, (%)	1,053 (33.61)
**Perceived potential applications of** **artificial intelligence in medicine/dentistry**	
Enhanced/automated medical diagnosis, (%)	1,804 (57.58)
Enhanced/automated disease prognosis, (%)	1,423 (45.42)
Optimized medical education, (%)	1,880 (60.01)
Optimized patient workflow, (%)	1,488 (47.49)
Nothing at all, (%)	96 (3.06)
**Concerns on artificial intelligence**	
Data privacy, (%)	1,144 (36.52)
Hacking and cybersecurity attacks, (%)	1,323 (42.23)
Fear of job replacement, (%)	1,209 (38.59)
Less human interaction with the patient, (%)	1,938 (61.86)

a *Individuals who responded either somewhat or strongly agree to the questions asking whether they self-regard as tech-savvy were considered tech-savvy*.

b *Countries were classified as developed or developing as defined by the United Nations (19)*.

*Data is presented as mean ± standard deviation or counts (%) as appropriate*.

**Table 2:** Survey results.

***n*** **= 3,133**	**Strongly disagree or equivalent**	**Somewhat disagree or equivalent**	**Undecided or equivalent**	**Somewhat agree or equivalent**	**Strongly agree or equivalent**	**Variance**	***P*** **-value (male vs. female)**	***P*** **-value (tech-savvy vs. non-tech-savvy)**	***P*** **-value (dental vs. medical student)**	***P*** **-value (pre-clinical vs. clinical)**	***P*** **-value (young vs. mature)**	***P*** **-value (developed vs. developing country)**
**Understanding on AI basic principles**												
Artificial intelligence is an umbrella term encompassing many technologies (e.g., “Machine Learning”). Do you have a basic understanding of these technologies?	195	743	1,340	536	219	0.969	**<0.0001**	**<0.0001**	0.438	**0.004**	0.879	**0.038**
Currently, AI has many applications in medicine (e.g., AI-assisted robotic surgery). How familiar are you with these applications?	515	847	1,075	510	186	1.228	**<0.0001**	**<0.0001**	0.570	**0.006**	0.790	**<0.0001**
Many applications we use in daily life already use AI (e.g., speech-/text-recognition, email spam-filters). How familiar are you with these applications?	133	347	989	1,073	591	1.103	**<0.0001**	**<0.0001**	0.757	**0.002**	**0.002**	**0.003**
**AI as a topic in medicine and dentistry**												
AI in medical research is rapidly evolving. Healthcare AI projects attracted more investment than any AI projects in any other field globally. How aware are you?	155	517	1,285	853	323	1.002	0.078	**<0.0001**	**0.021**	**<0.0001**	**0.003**	**<0.0001**
“Artificial Intelligence” and “Deep Learning” are currently being broadly discussed in the medical community. How aware of this are you?	173	623	1,222	807	308	1.050	0.057	**<0.0001**	**0.021**	**<0.0001**	**0.004**	**0.007**
To what extent do you feel you have an understanding of the technologies which underpin “Artificial Intelligence” and “Deep Learning”?	270	851	1,271	562	179	1.002	**<0.0001**	**<0.0001**	0.159	**<0.0001**	**0.012**	**0.0006**
**Attitudes and feelings toward AI**												
I perceive artificial intelligence in Medicine as a partner rather than as a competitor.	43	194	635	1,219	1,042	0.906	0.052	**<0.0001**	**0.002**	0.077	**0.024**	0.066
Artificial intelligence will revolutionize medicine/dentistry in general.	12	117	376	1,285	1,343	0.683	**0.008**	**0.005**	0.146	**0.017**	**0.0006**	**0.0004**
The non-interventional physician will be replaced in the foreseeable future.	388	780	950	794	221	1.270	0.394	**0.004**	**<0.0001**	**0.001**	**0.0007**	**<0.0001**
In the foreseeable future, all physicians will be replaced.	1,380	793	470	346	144	1.442	0.072	**0.028**	0.085	**0.002**	**0.0003**	**<0.0001**
These developments frighten me.	374	832	783	847	297	1.391	**<0.0001**	**<0.0001**	0.142	**<0.0001**	**0.004**	**<0.0001**
These developments make Medicine in general more exciting to me.	83	254	607	1,380	809	0.984	**0.003**	**<0.0001**	0.801	0.157	0.443	0.754
Artificial intelligence will never make the human physician expendable.	141	460	753	904	875	1.361	0.954	**0.015**	0.221	0.523	0.187	**0.046**
Artificial intelligence will improve Medicine in general.	17	78	272	1,352	1,414	0.598	**0.0004**	**<0.0001**	0.059	**0.014**	**0.011**	**0.005**
Artificial intelligence should be part of medical/dental training.	22	97	331	1,247	1,436	0.679	0.676	**0.037**	0.747	0.084	0.169	0.099

*All reported analyses used Mann-Whitney U-test. Significant p-values are highlighted in bold. For the directionality of the comparisons, please look at Supplementary Table 3 where odds ratios are presented*.

*AI, artificial intelligence*.

Regarding the sources of information on AI, most relied on web browsing (1,883 responses, 60.1%) and social media (1,861, 59.4%), while least relied on university (1,053, 33.6%) and friends or family (772, 24.6%). As regards the perceived applications of AI in Medicine and Dentistry, the most popular answers were medical education optimisation (1,880, 60.0%) and enhanced/automated medical diagnostic tasks (1,804, 57.6%). Conversely, the main concerns on the utilization of AI were the expected less human interaction with the patient (1,938, 61.9%) and any sensitive data leakage including cybersecurity attacks (1,323, 42.2%).

### Understanding on AI Basic Principles

Most students claimed at least a moderate understanding of the: (1) AI as an umbrella term encompassing many technologies (2,095, 67.9%), (2) current AI applications in medicine and dentistry (1,771, 56.5%); and (3) daily life AI applications (2,653, 84.9%). A higher agreement was associated with males (*p* < 0.0001), being tech-savvy (*p* < 0.0001), pre-clinical students (*p* < 0.006), and participants from a developed country (*p* < 0.04).

### AI as a Topic in Medicine and Dentistry

Most respondents were at least aware that AI is being broadly discussed in the medical community (2,337, 74.6%) and that healthcare AI projects attract more investment than any AI projects globally (2,461, 78.6%) (21). Higher rate of agreement was associated with tech-savvy respondents (*p* < 0.0001), medical students (*p* < 0.02), studies in a developing country (*p* < 0.007), pre-clinical students (*p* < 0.0001), and being 21 or younger (*p* < 0.004). When asked to indicate the specialty where AI will play the most decisive role in 20 years, most students answered surgery (1,146, 36.6%), followed by radiology (1,092, 34.9%).

### Attitudes and Feelings Toward AI

**Figure 1:**
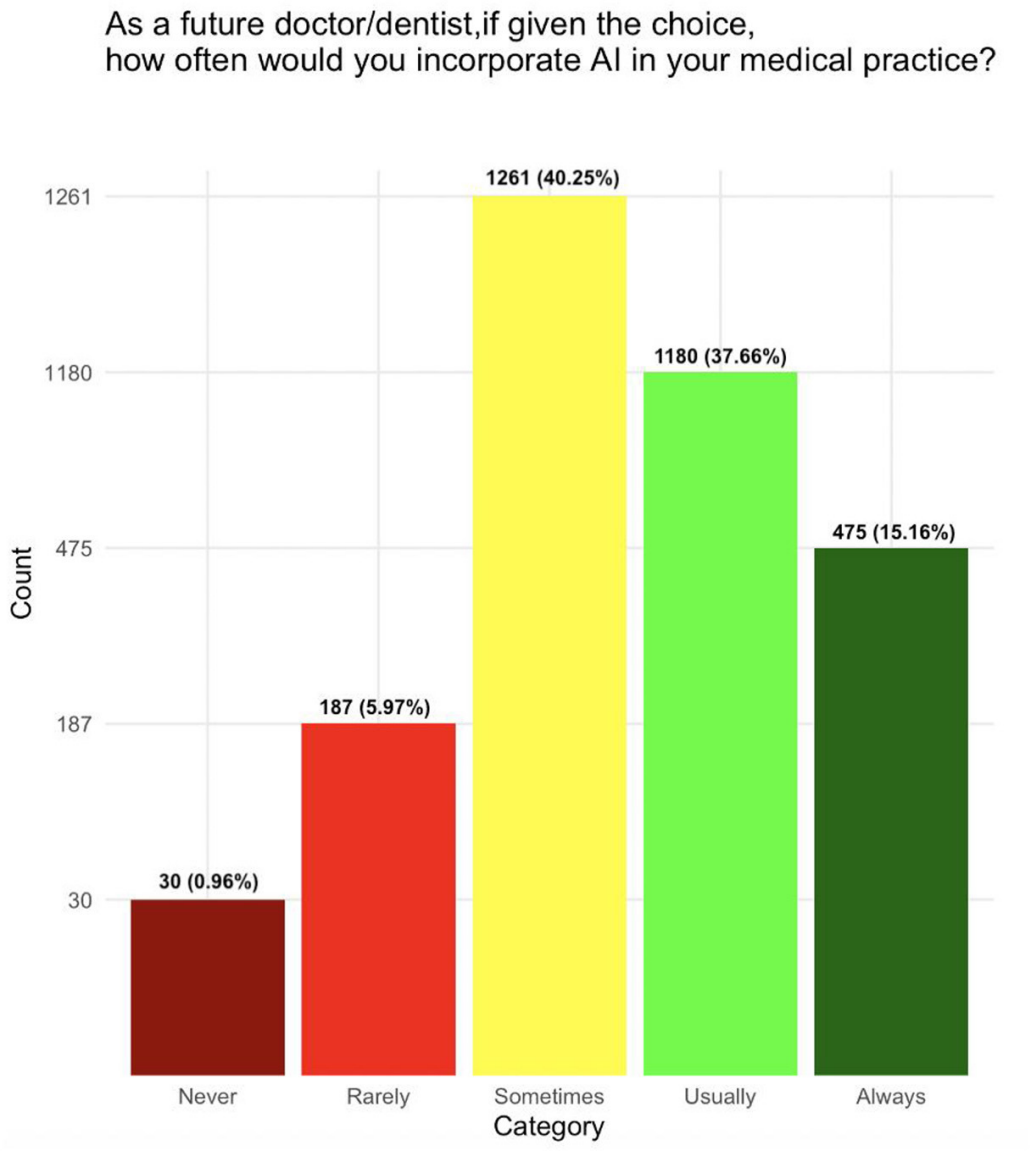
Inclination of medical and dental students to incorporate artificial intelligence in their future medical practice.

Students perceived AI as partner rather than as competitor (2,261, 72.2%) with a greater agreement rate observed for tech-savvy respondents (*p* < 0.0001) and medical students (*p* = 0.002). AI is believed among students to revolutionize medicine and dentistry (2,628, 83.9%), an opinion advocated strongly by males (*p* = 0.005) and students from a developed country (*p* = 0.0004). Interestingly, only the slight majority of the students agreed that AI will never make the human physician expendable (1,779, 56.8%). Furthermore, it was common belief in dental students (*p* = 0.001) and from respondents in developing countries (*p* < 0.0001) that non-interventional physician will be replaced. Most students agreed that the AI developments will make Medicine and Dentistry more exciting in the future (2,189, 69.9%) and that AI should be part of the core medical training curriculum (2,683, 85.6%). Higher agreement was only observed among tech-savvy respondents (*p* < 0.04). The slight majority of the respondents agreed that they would usually or always incorporate AI in their future practice (1,655, 52.8%) (Figure 1). The highest response variance was observed for “In the foreseeable future, all physicians will be replaced,” and “These developments frighten me, while the lowest variance was seen for “Artificial intelligence will improve Medicine in general,” and “Artificial intelligence should be part of medical/dental training.”

### Sensitivity Analyses

Binarizing survey responses to express disagreement (strongly or somewhat disagree, not at all, to a little extent, not at all or not so aware) or agreement (somewhat or agree, to a moderate or great extent or very great extent, and somewhat or very or extremely aware) was consistent with our initial analysis, with the exception of some results of borderline significance (Supplementary Table 2).

When pursuing adjustment for tech-savviness, the vast majority of associations persisted in the ordinal logistic regression models (Supplementary Table 3).

### Results by Country

A breakdown of survey responses for each country who had more than 50 respondents is provided in Supplementary Table 4.

## Discussion

### Principal Survey Findings

The data from this international multi-center survey study indicate that medical and dental students agree that AI developments make Medicine and Dentistry more exciting to them. They would like to see these developments implemented in their university curricula and plan to employ AI in their practice when they will graduate and qualify for their subspecialty.

### Key Survey Results

Firstly, most students have a basic understanding of the AI principles, are aware of the AI technologies they are already using and are up to date with the AI topics discussed in the medical community. Interestingly, this was particularly evident in pre-clinical students; this fact can be potentially attributed to their tendency to have higher exposure to science. Another possible explanation could be that as students get immersed with the clinical practice, their focus might be increasingly on the current scope and methods of clinical practice losing base with the latest research developments. Students from developed countries displayed higher agreement of having a better understanding of AI and its current developments, whereas our study states questions regarding the opportunity discrepancies among different country income categories indicating that students from developing countries might be at disadvantage. This further widens any existing global health inequalities (22). In support of this statement, is also the fact that there was a stronger majority among students from a developed country than from the lower income countries that AI will revolutionize medicine (Supplementary Table 4). Secondly, medical and dental students had mostly homogenous opinions except that medical students thought they were better informed on AI, while dental students were in stronger agreement that AI will replace human physicians. This is in keeping with the fact that their training is similar, and their fields are experiencing a similar exposure to AI. Thirdly, most students claim to use web-browsing as their primary source of information on AI. Self-initiated information gathering may be correlated with a better understanding and higher attraction to the AI. Only a third of respondents selected their university as a source of information on AI. This might reflect a level of satisfying exposure to AI education during their studies. Thus, our survey highlights an unaddressed need of incorporating AI into the medical and dental schools' curricula emerged from our investigation. Students would like namely to get more acquainted with AI and currently resort to internet sources to satisfy this demand. Similarly, most students (60.01%) also believe that AI could lead to an optimized medical education highlighting the room for improvement in the current teaching methods. Thus, it is not surprising that most students agreed that “Artificial intelligence should be part of medical/dental/training” and the variance of the survey item was one of the smallest. At the opposite end of the variance spectrum, the responses were widely distributed when the students were asked whether the AI developments frighten them, although most perceive AI as a partner rather than a competitor.

### Study Implications

AI in Medicine is undeniably an exciting prospect that could dramatically influence the next generation of professionals (23). AI interfaces using narrow and usually supervised machine learning can outperform under controlled circumstances certain medical specialties such as radiologists (24) and dermatologists (6). Medical and dental students are aware that AI is a hot topic in the domain, and most retrieve their information from web browsing or from social media. Currently, most students do not discern AI as a threat or fear job replacement. Nevertheless, they clearly desire to obtain ground-breaking knowledge in AI and be in pace with the latest developments. This could stem from the presumption that the doctors who are knowledgeable about AI will replace the ones who don't use it in this competitive field (25). Apparently, the medical and dental schools are lagging in providing their students with teaching of the basic AI principles and application, let alone providing technical support for basic machine learning experimental work. We feel that these results add to the empirical insights into the constraints of the current medical training and the appetite for reform. Nevertheless, it is little known whether medical schools are mature or, at least, prepared to embed a stronger emphasis on AI into the curricular medical training. Moreover, as the modern healthcare teams are multidisciplinary and include other professionals, e.g., nurses, clinical scientists, and pharmacists, it is ambiguous whether a cross-disciplinary approach is envisaged. Most respondents (61.86%) expressed concerns that as the role of AI will increase in Medicine and Dentistry, there could be less human interaction with the patient. Historically, the foundations of both professions have always been communication, empathy, and a close and caring relationship with the patient. Thus, these foundations should be persevered as AI immerses into clinical practice healthcare to avoid the loss of the human touch in the profession. However, a recent study highlighted that a lecture about AI reduced the students' concerns which suggests that further educational interventions are required to alleviate worries regarding AI (13).

Although students' perceptions are important, they might not be the ultimate determinant on how the medical curriculum is shaped and structured. Although it is a common practice in most universities to request feedback on the course from the students, it is unclear to what extent that feedback is acted upon. In addition, the primary factors affecting medical education have been postulated to be: social, technological, economic and political (26), and they exhibit a high geographical variation. Thus, it is unclear to what extent the universities' curricula are influenced by the desires of the medical and dental students. The inclusion of AI in the medical syllabus would also be a difficult task as it would be subjected to the policies of universities and national accreditation bodies. The medical students already tend to have a higher workload and be more distressed than their age-matched counterparts studying other degrees (27). To prevent further workload, certain aspects of the current curriculum might need to be removed to make room for AI. Further studies should explore what changes would be feasible. In addition, further research focusing on the attitudes of the academic staff, universities management and accreditation bodies on the utility and feasibility of incorporating AI into the medical curricula would provide more valuable insights.

### Strengths and Weaknesses

Although the ethics of Facebook is questionable (28), it has almost 3 billion monthly users as of 2021 and it is able to target a desired population given a set of characteristics. Even though its use in medical research is growing, it is still an underused tool. Our study is novel since it engaged a multinational community to explore the views of medical and dental students on AI using Facebook's targeting algorithm. The large number of study participants (*n* = 3,133) spanning 63 countries across all continents is the utmost strength of the study. In addition, the survey was designed sufficiently immune to fraudulent responses by obscuring attempts to submit multiple answers. However, the use of more sophisticated machinery such as virtual private networks cannot be confidently ruled out. The questions addressed covered a wide range of AI-related topics enabling us to explore the topic multidimensionally. We also collected enough demographics data to perform 6 relevant subgroup comparison analyses.

An important limitation of this work is that surveys may not be the best suited tools to conduct such exploratory work. In addition, currently there is no theoretical framework we could have used to guide the contents of our survey. Another impediment is the geographic heterogeneity of the responses. Although we have reached many medical and dental schools with a wide distribution on the world map, most of the students' national groups recorded <10 responses. In addition, within the countries with the highest number of responses, a limited number of medical schools was sampled. In addition, there may be concerns about the representativeness of our sample. Initially, we ran generic Facebook ads to medical and dental students without specifying any further characteristics (e.g., sex, age etc.). Then, we did advertising campaigns targeted to the groups who were under-represented in the first data collection sweep in order to match the sampled population with the medical/dental students population. However, Facebook itself is limited through its inherent non-probabilistic sampling as its machine learning algorithms targets individuals who are more likely to interact with an advertisement. This may have created bias toward tech savvy students who account for almost 80% of our sample. However, the associations persisted affect adjusting for tech-savviness. Please note that tech-savviness was self-rated and might not be a proxy of the respondents' actual technical capabilities. Similarly, the uneven distribution of answers between the countries can be also attributed to Facebook's sampling algorithm. This can affect the reproducibility of the study. In addition, we aimed for a female predominant sample, but there might be countries where males are over-represented as medical/dental students. The questions were selected to explore a wide range of attitudes and perceptions on AI, but they do not form a validated scale. However, the nature of the questions still enables us to infer certain conclusions about students' perception and stance on AI. Moreover, we decided to limit the number of questions asked to increase the number of participants. As self-assessments can be an unreliable proxy of actual knowledge (29), some of our survey questions may be prone to self-evaluation bias. In addition, data collection took place mostly at the beginning of the pandemic. Thus, many students might have had a temporarily elated view on AI brought by media reports portraying digital health as the future of Medicine. Although students raised concerns that AI could lead to less human interaction with the patients, our study did not explore whether the respondents are interested in AI because it would mean better care for their patients or merely because it would make the field more exciting to them.

### Study Position Compared to the Current Body of Evidence

A few other studies have explored the views of medical students on AI including a narrow investigation on specific subspecialties (10, 14, 30, 31). However, the novelty of our work is rooted in our focus on AI in both Medicine and Dentistry rather than in subspeciality such as radiology. In addition, to the best of our knowledge, we are the first to engage a multinational community of students across the globe. Lastly, we provided evidence elaborated at different demographic, geographic, school type (medical vs. dental), and studies level (pre-clinical vs. clinical) groups.

The AI in Medicine topic has ignited mixed feelings in the medical community, but the consensus is that the fate of the medical profession will change as AI gets immersed into the clinical practice (32). Although many see the advent of AI in clinical medicine as inevitable and advocate for its timely implementation in the medical schools' curricula (33), others are a bit skeptical and raised concerns on AI (34). Firstly, the privacy and control over data is ethically problematic (35). Secondly, there is a considerable heterogeneity between AI protocols in different centers (36). Thirdly, there are no standards for clinical care, quality, safety, and malpractice liability in the context of AI (34). Fourthly, there are instances where AI works extremely well [e.g., predicting schizophrenia onset (37)], but it is unclear how the algorithm makes its prediction [i.e., black box phenomenon (38)], making it difficult to rely on something we have no understanding on how it operates. Thus, AI might not be fully implemented in clinical practice very soon making the case for postponing incorporating AI into the medical curriculum.

## Conclusion

AI is undergoing a rapidly expanding role in medicine and dentistry. The next generation of medical and dental doctors perceive AI as a partner rather than a competitor and is planning to integrate it into their future practice. Thus, there might be a high demand to have AI topics integrated into the university curricula which should be further explored.

## Data Availability Statement

The raw data supporting the conclusions of this article will be made available by the authors, without undue reservation.

## Ethics Statement

Ethical review and approval was not required for the study on human participants in accordance with the local legislation and institutional requirements. The patients/participants provided their written informed consent to participate in this study.

## Author Contributions

SB, C-CT, and ZZ contributed significantly to the conceptualization, implementation, data acquisition, formal analysis, interpretation, and manuscript writing and are joint first authors. A-VI, LS, JS, M-MC, JL-R, DP, DA, CZ, AB, AL, LA-I, AS, AK, KM, EW, AO, DI, and EE contributed to the implementation, data acquisition, interpretation, and manuscript review. All authors contributed to the article and approved the submitted version.

## Conflict of Interest

The authors declare that the research was conducted in the absence of any commercial or financial relationships that could be construed as a potential conflict of interest.

## Publisher's Note

All claims expressed in this article are solely those of the authors and do not necessarily represent those of their affiliated organizations, or those of the publisher, the editors and the reviewers. Any product that may be evaluated in this article, or claim that may be made by its manufacturer, is not guaranteed or endorsed by the publisher.

## Abbreviations

AI: artificial intelligence.
