# Supplementary material for: Artificial Intelligence in Medicine: A Multinational Multi-Center Survey on the Medical and Dental Students' Perception
Source: Front Public Health. 2021 Dec 24;9:795284. doi: 10.3389/fpubh.2021.795284 (PMC8739771; doi:10.3389/fpubh.2021.795284)
Supplement: Supplementary file 1 [file Table_1.pdf]

ADDITIONAL FILE FOR:

Artificial Intelligence in Medicine: A Multinational Multi-Centre Survey on the Medical and Dental Students' Perception

Authors:

Sotirios Bisdas[1,2*], Constantin-Cristian Topriceanu[3*], Zosia Zakrzewska[3*], Alexandra-Valentina Irimia[4], Loizos Shakallis [1], Jithu Subhash[5], Maria-Madalina Casapu[6], Jose Leon-Rojas [7], Daniel Pinto dos Santos [8], Dilys Miriam Andrews [9], Claudia Zeicu [10], Ahmad Mohammad Bouhuwaish[11], Avinindita Nura Lestari[12], Abdalkadir Abu-Ismail [13], Arsal Subbah Sadiq[14], Almu'atasim D'yab Khamees [13], Khaled MG Mohammed[15], Estelle Williams[16], Aya Ibrahim Omran[11], Dima Abu Ismail [17], Esraa Hasan Ebrahim[18]

*Joint 1st authors.

Author Affiliations:

1. Department of Neuroradiology, The National Hospital for Neurology and Neurosurgery, University College London NHS Foundation Trust, London, UK
2. Department of Brain Repair & Rehabilitation, Queen Square Institute of Neurology, University College London, London, UK
3. University College London Medical School, London, United Kingdom
4. Computer Science Department, University College London, London, UK
5. School of Medicine, Nottingham University, Nottingham, United Kingdom
6. Carol Davila University of Medicine and Pharmacy, Bucharest, Romania
7. International University of Ecuador, Quito, Ecuador Universidad Internacional del Ecuador, NeurALL Research Group, School of Medicine, Quito, Ecuador.
8. University of Cologne, Cologne, Germany

9. School of Medicine, Cardiff University, Cardiff, United Kingdom
10. Department of Clinical Neurophysiology, The National Hospital for Neurology and Neurosurgery, University College London NHS Foundation Trust, London, UK
11. Faculty of Medicine, University of Tobruk, Tripoli, Libya
12. Universitas Islam Bandung, Bandung, Indonesia
13. Yarmouk University, Irbid, Jordan
14. CMH Medical College Lahore, Lahore, Pakistan
15. Tanta University, Tanta, Egypt
16. Peninsula Dental School, University of Plymouth, Plymouth, United Kingdom
17. Hashemite University, Zarqua, Jordan
18. Sabha University, Sabha, Libya

Corresponding author:

Sotirios Bisdas

Department of Neuroradiology, 8-11 Queen Square, Box 65, London WC1N 3BG, United Kingdom

Tel: 0044 20 344 83148

Email: s.bisdas@ucl.ac.uk

Additional Table A1. Country of study for the respondents.

Country	Continent	Number of respondents
Albania	Europe	5
Algeria	Africa	18
Australia	Australia	4
Bangladesh	Asia	1
Belarus	Europe	1
Brazil	South America	5
Bulgaria	Europe	2
Canada	North America	3
China	Asia	1
Croatia	Europe	12
Cuba	North America	3
Cyprus	Europe	93
Czech Republic	Europe	1
Ecuador	South America	200
Egypt	Africa/Asia	156
France	Europe	59
Germany	Europe	50
Greece	Europe	1
Grenada	North America	1
Guatemala	North America	1
Hong Kong	Asia	1
Hungary	Europe	1
India	Asia	5
Indonesia	Asia	288
Iraq	Asia	12
Ireland	Europe	12
Italy	Europe	11
Jordan	Asia	455
Lebanon	Asia	1
Libya	Africa	790
Lithuania	Europe	1
Malaysia	Asia	2
Malta	Europe	3
Mexico	North America	3
Morocco	Africa	7
Myanmar	Asia	3
Netherlands	Europe	1
Norway	Europe	2
Oman	Asia	65
Pakistan	Asia	160
Palestine	Asia	5

Peru	South America	5
Poland	Europe	34
Qatar	Asia	2
Romania	Europe	186
Russia	Europe/Asia	2
Saudi Arabia	Asia	1
Serbia	Europe	7
Slovakia	Europe	4
Slovenia	Europe	1
South Africa	Africa	46
Sri Lanka	Asia	1
Sudan	Africa	6
Sweden	Europe	14
Syria	Asia	7
Tunisia	Africa	2
Turkey	Asia/Europe	18
Uganda	Africa	1
UK	Europe	331
Ukraine	Europe	2
USA	North America	4
Venezuela	South America	7
Yemen	Asia	7

Additional Table A2. Survey results when responses were binarized to express disagreement (strongly or somewhat disagree, not at all, to a little extent, not at all or not so aware) or agreement (somewhat or agree, to a moderate or great extent or very great extent, and somewhat or very or extremely aware).

	p-value (male vs female)	p-value (tech-savvy vs non-tech-savvy)	p-value (dental vs medical student)	p-value (pre-clinical vs clinical)	p-value (young vs mature	p-value (developed vs developing country)
**Subsection 2: Understanding on AI basic principles**						
Artificial intelligence is an umbrella term encompassing many technologies (e.g. 'Machine Learning'). Do you have a basic understanding of these technologies?	**<0.0001**	**<0.0001**	0.962	**0.029**	0.547	0.491*
Currently, AI has many applications in Medicine (e.g. AI-assisted robotic surgery). How familiar are you with these applications?	**<0.0001**	**<0.0001**	0.482	**0.004**	0.580	**<0.0001**
Many applications we use in daily life already use AI (e.g. speech-/text-recognition, email spam-filters). How familiar are you with these applications?	**<0.0001**	**<0.0001**	0.829	0.056*	0.348*	**0.002**
**Subsection 3: AI as a topic in Medicine and Dentistry**						
AI in medical research is rapidly evolving. Healthcare AI projects attracted more investment than any AI projects in any other field globally. How aware are you?	0.953	**<0.0001**	**0.011**	**<0.0001**	**0.023**	**<0.0001**
‘Artificial Intelligence’ and ‘Deep Learning’ are currently being broadly discussed in the medical community. How aware of this are you?	0.265	**<0.0001**	0.210*	**0.0005**	0.213*	**<0.0001**
To what extent do you feel you have an understanding of the technologies which underpin ‘Artificial Intelligence’ and ‘Deep Learning’?	**<0.0001**	**<0.0001**	0.061	**<0.0001**	**0.016**	**0.001**

Subsection 4: Attitudes and feelings towards AI						
I perceive AI in Medicine as a partner rather than as a competitor.	0.500	**<0.0001**	**0.030**	0.371	0.069*	0.548
Artificial intelligence will revolutionize Medicine/Dentistry in general.	0.902*	0.063*	1.00	1.00*	0.200*	0.081*
The non-interventional physician will be replaced in the foreseeable future.	0.355	**0.011**	**<0.0001**	**0.005**	**0.0007**	**<0.0001**
In the foreseeable future, all physicians will be replaced.	**0.038***	**0.020**	0.570	**0.002**	**0.003**	**<0.0001**
These developments frighten me.	**0.003**	**<0.0001**	0.204	**<0.0001**	**0.006**	**<0.0001**
These developments make Medicine in general more exciting to me.	**0.010**	**<0.0001**	0.529	0.493	0.421	0.666
Artificial intelligence will never make the human physician expendable.	0.730	**0.002**	0.423	0.113	0.531	**0.007**
Artificial intelligence will improve Medicine in general.	**0.410***	**0.0005**	0.604	0.806*	0.471*	**0.034**
Artificial intelligence should be part of medical/dental training.	0.731	**0.329***	1.00	1.00	0.942	0.613

* The sensitivity analysis yielded a p-value which has crossed either way the significance level of 0.05.

All reported analyses used Pearson’s Chi-squared test with Yates’ continuity correction. Significant *p*-values are highlighted in bold. For the directionality of the results please view Additional Table 3.

AI, artificial intelligence.

Additional Table A3. Survey results when further adjustment for tech-savviness was pursued.

	Male vs Female[1]			Dental vs Medical[2]			Pre-clinical vs clinical[3]			Young vs mature[4]			Developed vs developing country[5]		
**Subsection 2: Understanding on AI basic principles**	**OR (95% CI)**	**p-value**	**Brant p-value**	**OR (95% CI)**	**p-value**	**Brant p-value**	**OR (95% CI)**	**p-value**	**Brant p-value**	**OR (95% CI)**	**p-value**	**Brant p-value**	**OR (95% CI)**	**p-value**	**Brant p-value**
Artificial intelligence is an umbrella term encompassing many technologies (e.g. 'Machine Learning'). Do you have a basic understanding of these technologies?	1.64 (1.43, 5.20)	**<0.0001**	0.14	1.02 (0.87, 1.20)	0.768	0.05	0.82 (0.72, 0.94)	**0.004**	0.09	1.02 (0.90, 1.17)	0.713	0.12	1.05 (0.90, 1.21)	0.543*	**0.01**
Currently, AI has many applications in Medicine (e.g. AI-assisted robotic surgery). How familiar are you with these applications?	1.56 (1.37, 1.79)	**<0.0001**	0.150	0.92 (0.79, 1.08)	0.327	**0.002**	0.84 (0.74, 0.96)	**0.009**	**0.003**	0.99 (0.88, 1.13)	0.906	0.095	1.33 (1.16, 1.54)	**<0.0001**	**0.005**
Many applications we use in daily life already use AI (e.g. speech-/text-recognition, email spam-filters). How familiar are you with these applications?	1.40 (1.22, 1.60)	**<0.0001**	0.190	0.99 (0.85, 1.16)	0.907	0.323	0.81 (0.72, 0.93)	**0.002**	0.295	0.82 (0.73, 0.94)	**0.003**	0.115	1.15 (0.99, 1.33)	0.060*	0.222
**Subsection 3: AI as a topic in Medicine and Dentistry**															
AI in medical research is rapidly evolving. Healthcare AI projects attracted more investment than any AI projects in any other field	1.06 (0.92, 1.21)	0.429	0.148	0.81 (0.69, 0.95)	**0.010**	0.371	0.70 (0.59, 0.76)	**<0.0001**	0.667	0.83 (0.73, 0.95)	**0.005**	0.642	0.64 (0.55, 0.74)	**<0.0001**	**0.0005**

globally. How aware are you?															
‘Artificial Intelligence’ and ‘Deep Learning’ are currently being broadly discussed in the medical community. How aware of this are you?	1.05 (0.91, 1.20)	0.521	0.935	0.81 (0.70, 0.95)	**0.010**	0.962	0.70 (0.62, 0.80)	**<0.0001**	0.355	0.84 (0.74, 0.96)	**0.008**	0.471	0.75 (0.65, 0.87)	**0.0002**	**0.005**
To what extent do you feel you have an understanding of the technologies which underpin ‘Artificial Intelligence’ and ‘Deep Learning’?	1.29<br><br>(1.13, 1.48)	**0.0003**	0.341	0.86 (0.74, 1.02)	0.075	**0.047**	0.63 (0.55, 0.72)	**<0.0001**	0.488	0.86 (0.76, 0.98)	**0.022**	0.390	0.68 (0.59, 0.79)	**<0.0001**	**0.0001**
**Subsection 4: Attitudes and feelings towards AI**															
I perceive AI in Medicine as a partner rather than as a competitor.	1.08 (0.94, 1.24)	0.267	0.067	0.77 (0.65, 0.90)	**0.001**	0.157	0.89 (0.78, 1.02)	0.091	0.336	0.87 (0.77, 0.99)	**0.036**	0.247	1.11 (0.96, 1.28)	0.181	0.357
Artificial intelligence will revolutionize Medicine/Dentistry in general.	1.19 (1.03, 1.37)	**0.018**	**0.043**	0.88 (0.75, 1.04)	0.132	0.093	0.85 (0.74, 0.97)	**0.017**	0.138	0.80 (0.70, 0.91)	**0.0007**	0.071	1.29 (1.11, 1.50)	**0.001**	**0.042**
The non-interventional physician will be replaced in the foreseeable future.	1.24 (1.06, 1.45)	0.579	0.340	1.42 (1.21, 1.66)	**<0.0001**	0.336	0.81 (0.71, 0.92)	**0.002**	0.292	0.81 (0.71, 0.92)	**0.0009**	0.062	0.67 (0.58, 0.78)	**<0.0001**	0.087
In the foreseeable future, all physicians will be replaced.	1.12 (0.97, 1.28)	0.116	0.516	1.15 (0.98, 1.34)	0.092	0.225	0.82 (0.71, 0.93)	**0.002**	0.228	0.79 (0.69, 0.90)	**0.0003**	0.474	0.61 (0.53, 0.71)	**<0.0001**	0.150
These developments frighten me.	0.76 (0.66, 0.87)	**<0.0001**	**<0.0001**	0.90 (0.77, 1.05)	0.178	0.189	0.67 (0.59, 0.76)	**<0.0001**	0.089	0.83 (0.73, 0.94)	**0.003**	0.044	0.45 (0.39, 0.52)	**<0.0001**	**<0.0001**
These developments make Medicine in general more exciting to me.	1.16 (1.01, 1.33)	**0.040**	0.262	0.96 (0.82, 1.13)	0.622	0.447	0.91 (0.80, 1.04)	0.162	0.199	0.96 (0.84, 1.09)	0.538	0.077	0.93 (0.80, 1.08)	0.336	0.668
Artificial intelligence will never make the	0.99 (0.86, 1.13)	0.830	0.395	0.91 (0.78, 1.05)	0.199	**0.010**	1.04 (0.92, 1.19)	0.522	**0.047**	1.09 (0.96, 1.24)	0.176	0.419	1.14 (0.99, 1.31)	0.069	0.090

human physician expendable.															
Artificial intelligence will improve Medicine in general.	1.26 (1.09, 1.45)	**0.002**	0.285	0.85 (0.72, 1.00)	**0.047**	0.260	0.85 (0.74, 0.97)	**0.016**	0.511	0.85 (0.74, 0.97)	**0.013**	0.380	1.22 (1.04, 1.41)	**0.012**	0.224
Artificial intelligence should be part of medical/dental training.	0.95 (0.83, 1.10)	0.505	**0.044**	0.97 (0.82, 1.14)	0.706	0.071	0.89 (0.78, 1.02)	0.088	0.119	0.91 (0.80, 1.04)	0.177	0.081	0.87 (0.75, 1.01)	0.068	**0.028**

* The sensitivity analysis yielded a p-value which has crossed either way the significance level of 0.05.

All reported analyses used generalized linear models with ordinal logit link (i.e., ordinal logistic regression). Significant *p*-values are highlighted in bold. Adjustment was made for tech-savviness.

[1] Males were coded as 1, while females were coded as 0.
[2] Dental students were coded as 1, while medical students were coded as 0.
[3] Clinical students were coded as 1, while pre-clinical students were coded as 0.
[4] Mature students were coded as 1, while young students were coded as 0.
[5] Students from developed countries were coded as 1, while students from developing countries were coded as 0.

Additional Table A4. Survey results by country.

	Strongly Disagree or equivalent	Somewhat Disagree or equivalent	Undecided or equivalent	Somewhat Agree or equivalent	Strongly Agree or equivalent
**CYPRUS (n=93)**					
**Understanding on AI basic principles**					
Artificial intelligence is an umbrella term encompassing many technologies (e.g., 'Machine Learning'). Do you have a basic understanding of these technologies?	5	17	42	25	4
Currently, AI has many applications in Medicine (e.g., AI-assisted robotic surgery). How familiar are you with these applications?	12	27	33	18	3
Many applications we use in daily life already use AI (e.g. speech-/text-recognition, email spam-filters). How familiar are you with these applications?	4	4	21	34	30
**AI as a topic in Medicine and Dentistry**					
AI in medical research is rapidly evolving. Healthcare AI projects attracted more investment than any AI projects in any other field globally. How aware are you?	8	26	30	25	4

‘Artificial Intelligence’ and ‘Deep Learning’ are currently being broadly discussed in the medical community. How aware of this are you?	5	30	30	22	6
To what extent do you feel you have an understanding of the technologies which underpin ‘Artificial Intelligence’ and ‘Deep Learning’?	8	28	41	14	2
**Attitudes and feelings towards AI**					
I perceive Artificial Intelligence in Medicine as a partner rather than as a competitor.	1	1	15	45	41
Artificial intelligence will revolutionize Medicine/Dentistry in general.	0	2	8	30	53
The non-interventional physician will be replaced in the foreseeable future.	17	31	22	17	6
In the foreseeable future, all physicians will be replaced.	47	27	12	7	0
These developments frighten me.	23	22	24	24	0
These developments make Medicine in general more exciting to me.	1	9	20	44	19
Artificial intelligence will never make the human physician expendable.	1	13	24	34	21
Artificial intelligence will improve Medicine in general.	0	0	5	44	44
Artificial intelligence should be part of medical/dental training.	0	1	10	30	52

ECUADOR (N=200)					
**Understanding on AI basic principles**					
Artificial intelligence is an umbrella term encompassing many technologies (e.g., 'Machine Learning'). Do you have a basic understanding of these technologies?	15	56	83	40	6
Currently, AI has many applications in Medicine (e.g., AI-assisted robotic surgery). How familiar are you with these applications?	39	50	78	26	7
Many applications we use in daily life already use AI (e.g. speech-/text-recognition, email spam-filters). How familiar are you with these applications?	5	17	51	76	51
**AI as a topic in Medicine and Dentistry**					
AI in medical research is rapidly evolving. Healthcare AI projects attracted more investment than any AI projects in any other field globally. How aware are you?	18	44	67	49	22
‘Artificial Intelligence’ and ‘Deep Learning’ are currently being broadly discussed in the medical community. How aware of this are you?	17	42	63	62	16
To what extent do you feel you have an understanding of the technologies which	26	52	81	32	9

underpin ‘Artificial Intelligence’ and ‘Deep Learning’?					
**Attitudes and feelings towards AI**					
I perceive Artificial Intelligence in Medicine as a partner rather than as a competitor.	2	10	46	77	65
Artificial intelligence will revolutionize Medicine/Dentistry in general.	0	2	16	54	128
The non-interventional physician will be replaced in the foreseeable future.	23	45	62	57	13
In the foreseeable future, all physicians will be replaced.	69	52	30	37	12
These developments frighten me.	39	57	38	51	15
These developments make Medicine in general more exciting to me.	4	14	43	82	57
Artificial intelligence will never make the human physician expendable.	5	15	47	59	74
Artificial intelligence will improve Medicine in general.	1	1	11	70	117
Artificial intelligence should be part of medical/dental training.	0	7	7	74	112

EGYPT (n=156)					
**Understanding on AI basic principles**					
Artificial intelligence is an umbrella term encompassing many technologies (e.g., 'Machine Learning'). Do you have a basic understanding of these technologies?	7	37	78	29	5
Currently, AI has many applications in Medicine (e.g., AI-assisted robotic surgery). How familiar are you with these applications?	30	54	55	15	2
Many applications we use in daily life already use AI (e.g. speech-/text-recognition, email spam-filters). How familiar are you with these applications?	10	21	62	46	17
**AI as a topic in Medicine and Dentistry**					
AI in medical research is rapidly evolving. Healthcare AI projects attracted more investment than any AI projects in any other field globally. How aware are you?	7	15	72	51	11
'Artificial Intelligence' and 'Deep Learning' are currently being broadly discussed in the medical community. How aware of this are you?	4	27	66	45	13
To what extent do you feel you have an understanding of the technologies which	12	55	64	22	3

underpin 'Artificial Intelligence' and 'Deep Learning'?					
**Attitudes and feelings towards AI**					
I perceive Artificial Intelligence in Medicine as a partner rather than as a competitor.	1	14	33	68	40
Artificial intelligence will revolutionize Medicine/Dentistry in general.	2	2	26	62	64
The non-interventional physician will be replaced in the foreseeable future.	20	45	51	34	6
In the foreseeable future, all physicians will be replaced.	84	41	21	8	2
These developments frighten me.	1	37	47	54	17
These developments make Medicine in general more exciting to me.	2	19	31	84	20
Artificial intelligence will never make the human physician expendable.	11	28	39	36	42
Artificial intelligence will improve Medicine in general.	0	5	19	81	51
Artificial intelligence should be part of medical/dental training.	0	4	18	80	54

FRANCE (n=59)					
**Understanding on AI basic principles**					
Artificial intelligence is an umbrella term encompassing many technologies (e.g., 'Machine Learning'). Do you have a basic understanding of these technologies?	2	4	17	24	12
Currently, AI has many applications in Medicine (e.g., AI-assisted robotic surgery). How familiar are you with these applications?	2	8	13	18	18
Many applications we use in daily life already use AI (e.g. speech-/text-recognition, email spam-filters). How familiar are you with these applications?	2	3	16	28	10
**AI as a topic in Medicine and Dentistry**					
AI in medical research is rapidly evolving. Healthcare AI projects attracted more investment than any AI projects in any other field globally. How aware are you?	2	2	15	26	14
'Artificial Intelligence' and 'Deep Learning' are currently being broadly discussed in the medical community. How aware of this are you?	2	7	15	22	13
To what extent do you feel you have an understanding of the technologies which	2	5	19	24	9

underpin 'Artificial Intelligence' and 'Deep Learning'?					
**Attitudes and feelings towards AI**					
I perceive Artificial Intelligence in Medicine as a partner rather than as a competitor.	0	1	9	28	21
Artificial intelligence will revolutionize Medicine/Dentistry in general.	1	0	12	21	25
The non-interventional physician will be replaced in the foreseeable future.	5	12	19	18	5
In the foreseeable future, all physicians will be replaced.	16	19	14	6	4
These developments frighten me.	11	16	13	11	8
These developments make Medicine in general more exciting to me.	1	8	10	23	17
Artificial intelligence will never make the human physician expendable.	1	7	25	16	10
Artificial intelligence will improve Medicine in general.	1	1	4	22	31
Artificial intelligence should be part of medical/dental training.	0	1	11	16	31

GERMANY (n=50)					
**Understanding on AI basic principles**					
Artificial intelligence is an umbrella term encompassing many technologies (e.g., 'Machine Learning'). Do you have a basic understanding of these technologies?	2	14	23	7	4
Currently, AI has many applications in Medicine (e.g., AI-assisted robotic surgery). How familiar are you with these applications?	6	18	15	4	7
Many applications we use in daily life already use AI (e.g. speech-/text-recognition, email spam-filters). How familiar are you with these applications?	1	9	14	14	12
**AI as a topic in Medicine and Dentistry**					
AI in medical research is rapidly evolving. Healthcare AI projects attracted more investment than any AI projects in any other field globally. How aware are you?	2	11	17	13	7
'Artificial Intelligence' and 'Deep Learning' are currently being broadly discussed in the medical community. How aware of this are you?	2	3	23	13	9
To what extent do you feel you have an understanding of the technologies which	2	12	23	8	5

underpin ‘Artificial Intelligence’ and ‘Deep Learning’?					
**Attitudes and feelings towards AI**					
I perceive Artificial Intelligence in Medicine as a partner rather than as a competitor.	0	5	5	18	22
Artificial intelligence will revolutionize Medicine/Dentistry in general.	0	2	5	25	18
The non-interventional physician will be replaced in the foreseeable future.	14	11	10	11	4
In the foreseeable future, all physicians will be replaced.	28	8	4	2	8
These developments frighten me.	10	13	7	17	3
These developments make Medicine in general more exciting to me.	0	6	10	24	10
Artificial intelligence will never make the human physician expendable.	1	4	7	18	20
Artificial intelligence will improve Medicine in general.	0	1	2	21	26
Artificial intelligence should be part of medical/dental training.	0	1	5	22	22

INDONESIA (n=288)					
**Understanding on AI basic principles**					
Artificial intelligence is an umbrella term encompassing many technologies (e.g., 'Machine Learning'). Do you have a basic understanding of these technologies?	28	96	114	41	9
Currently, AI has many applications in Medicine (e.g., AI-assisted robotic surgery). How familiar are you with these applications?	55	118	76	34	5
Many applications we use in daily life already use AI (e.g. speech-/text-recognition, email spam-filters). How familiar are you with these applications?	8	32	89	101	58
**AI as a topic in Medicine and Dentistry**					
AI in medical research is rapidly evolving. Healthcare AI projects attracted more investment than any AI projects in any other field globally. How aware are you?	7	46	147	65	23
'Artificial Intelligence' and 'Deep Learning' are currently being broadly discussed in the medical community. How aware of this are you?	11	62	125	71	19
To what extent do you feel you have an understanding of the technologies which	14	109	118	41	6

underpin ‘Artificial Intelligence’ and ‘Deep Learning’?					
**Attitudes and feelings towards AI**					
I perceive Artificial Intelligence in Medicine as a partner rather than as a competitor.	2	13	52	135	86
Artificial intelligence will revolutionize Medicine/Dentistry in general.	0	10	37	156	85
The non-interventional physician will be replaced in the foreseeable future.	21	73	104	73	17
In the foreseeable future, all physicians will be replaced.	95	104	50	32	7
These developments frighten me.	36	105	65	69	13
These developments make Medicine in general more exciting to me.	0	15	52	146	75
Artificial intelligence will never make the human physician expendable.	9	58	87	95	39
Artificial intelligence will improve Medicine in general.	0	3	10	161	114
Artificial intelligence should be part of medical/dental training.	1	3	32	144	108

JORDAN (n=455)					
**Understanding on AI basic principles**					
Artificial intelligence is an umbrella term encompassing many technologies (e.g., 'Machine Learning'). Do you have a basic understanding of these technologies?	25	106	167	107	50
Currently, AI has many applications in Medicine (e.g., AI-assisted robotic surgery). How familiar are you with these applications?	51	103	143	108	50
Many applications we use in daily life already use AI (e.g. speech-/text-recognition, email spam-filters). How familiar are you with these applications?	11	28	108	175	133
**AI as a topic in Medicine and Dentistry**					
AI in medical research is rapidly evolving. Healthcare AI projects attracted more investment than any AI projects in any other field globally. How aware are you?	9	45	190	149	62
'Artificial Intelligence' and 'Deep Learning' are currently being broadly discussed in the medical community. How aware of this are you?	13	65	164	148	65
To what extent do you feel you have an understanding of the technologies which	14	94	181	114	52

underpin 'Artificial Intelligence' and 'Deep Learning'?					
**Attitudes and feelings towards AI**					
I perceive Artificial Intelligence in Medicine as a partner rather than as a competitor.	5	22	66	199	163
Artificial intelligence will revolutionize Medicine/Dentistry in general.	2	15	56	187	195
The non-interventional physician will be replaced in the foreseeable future.	43	88	135	138	51
In the foreseeable future, all physicians will be replaced.	187	92	74	69	33
These developments frighten me.	51	106	103	139	56
These developments make Medicine in general more exciting to me.	10	39	77	185	144
Artificial intelligence will never make the human physician expendable.	18	65	100	138	134
Artificial intelligence will improve Medicine in general.	1	13	36	181	224
Artificial intelligence should be part of medical/dental training.	3	16	40	175	221

LIBYA (n=790)					
**Understanding on AI basic principles**					
Artificial intelligence is an umbrella term encompassing many technologies (e.g., 'Machine Learning'). Do you have a basic understanding of these technologies?	60	149	370	156	55
Currently, AI has many applications in Medicine (e.g., AI-assisted robotic surgery). How familiar are you with these applications?	180	187	265	126	32
Many applications we use in daily life already use AI (e.g. speech-/text-recognition, email spam-filters). How familiar are you with these applications?	55	112	278	253	92
**AI as a topic in Medicine and Dentistry**					
AI in medical research is rapidly evolving. Healthcare AI projects attracted more investment than any AI projects in any other field globally. How aware are you?	31	101	323	226	109
'Artificial Intelligence' and 'Deep Learning' are currently being broadly discussed in the medical community. How aware of this are you?	38	130	344	184	94
To what extent do you feel you have an understanding of the technologies which	51	183	361	150	45

underpin ‘Artificial Intelligence’ and ‘Deep Learning’?					
**Attitudes and feelings towards AI**					
I perceive Artificial Intelligence in Medicine as a partner rather than as a competitor.	17	61	183	253	276
Artificial intelligence will revolutionize Medicine/Dentistry in general.	6	50	105	311	318
The non-interventional physician will be replaced in the foreseeable future.	105	202	243	181	59
In the foreseeable future, all physicians will be replaced.	357	195	106	84	48
These developments frighten me.	5	208	222	238	117
These developments make Medicine in general more exciting to me.	35	77	151	322	205
Artificial intelligence will never make the human physician expendable.	55	123	184	184	244
Artificial intelligence will improve Medicine in general.	12	31	103	305	339
Artificial intelligence should be part of medical/dental training.	10	34	90	278	378

OMAN (n=65)					
**Understanding on AI basic principles**					
Artificial intelligence is an umbrella term encompassing many technologies (e.g., 'Machine Learning'). Do you have a basic understanding of these technologies?	0	19	36	6	4
Currently, AI has many applications in Medicine (e.g., AI-assisted robotic surgery). How familiar are you with these applications?	8	17	35	5	0
Many applications we use in daily life already use AI (e.g. speech-/text-recognition, email spam-filters). How familiar are you with these applications?	2	11	28	18	6
**AI as a topic in Medicine and Dentistry**					
AI in medical research is rapidly evolving. Healthcare AI projects attracted more investment than any AI projects in any other field globally. How aware are you?	1	13	35	14	2
'Artificial Intelligence' and 'Deep Learning' are currently being broadly discussed in the medical community. How aware of this are you?	4	21	27	10	3
To what extent do you feel you have an understanding of the technologies which	4	27	26	7	1

underpin 'Artificial Intelligence' and 'Deep Learning'?					
**Attitudes and feelings towards AI**					
I perceive Artificial Intelligence in Medicine as a partner rather than as a competitor.	1	2	12	34	16
Artificial intelligence will revolutionize Medicine/Dentistry in general.	1	3	17	16	28
The non-interventional physician will be replaced in the foreseeable future.	4	20	28	11	2
In the foreseeable future, all physicians will be replaced.	41	13	8	2	1
These developments frighten me.	8	16	16	18	7
These developments make Medicine in general more exciting to me.	1	4	26	21	13
Artificial intelligence will never make the human physician expendable.	4	11	14	18	18
Artificial intelligence will improve Medicine in general.	0	1	5	31	28
Artificial intelligence should be part of medical/dental training.	0	1	10	22	32

PAKISTAN (n=160)					
**Understanding on AI basic principles**					
Artificial intelligence is an umbrella term encompassing many technologies (e.g., 'Machine Learning'). Do you have a basic understanding of these technologies?	7	46	70	31	6
Currently, AI has many applications in Medicine (e.g., AI-assisted robotic surgery). How familiar are you with these applications?	37	44	57	21	1
Many applications we use in daily life already use AI (e.g. speech-/text-recognition, email spam-filters). How familiar are you with these applications?	10	27	51	47	25
**AI as a topic in Medicine and Dentistry**					
AI in medical research is rapidly evolving. Healthcare AI projects attracted more investment than any AI projects in any other field globally. How aware are you?	10	47	73	26	4
'Artificial Intelligence' and 'Deep Learning' are currently being broadly discussed in the medical community. How aware of this are you?	9	51	72	23	5
To what extent do you feel you have an understanding of the technologies which	16	62	64	14	4

underpin ‘Artificial Intelligence’ and ‘Deep Learning’?					
**Attitudes and feelings towards AI**					
I perceive Artificial Intelligence in Medicine as a partner rather than as a competitor.	2	11	41	77	29
Artificial intelligence will revolutionize Medicine/Dentistry in general.	0	7	17	86	50
The non-interventional physician will be replaced in the foreseeable future.	10	19	44	71	16
In the foreseeable future, all physicians will be replaced.	44	39	34	35	8
These developments frighten me.	20	34	48	46	12
These developments make Medicine in general more exciting to me.	7	15	20	88	30
Artificial intelligence will never make the human physician expendable.	5	17	46	64	28
Artificial intelligence will improve Medicine in general.	0	6	19	87	48
Artificial intelligence should be part of medical/dental training.	1	5	21	72	61

ROMANIA (n=186)					
**Understanding on AI basic principles**					
Artificial intelligence is an umbrella term encompassing many technologies (e.g., 'Machine Learning'). Do you have a basic understanding of these technologies?	7	47	83	36	13
Currently, AI has many applications in Medicine (e.g., AI-assisted robotic surgery). How familiar are you with these applications?	24	45	73	38	6
Many applications we use in daily life already use AI (e.g. speech-/text-recognition, email spam-filters). How familiar are you with these applications?	5	20	63	62	36
**AI as a topic in Medicine and Dentistry**					
AI in medical research is rapidly evolving. Healthcare AI projects attracted more investment than any AI projects in any other field globally. How aware are you?	7	25	86	55	13
‘Artificial Intelligence’ and ‘Deep Learning’ are currently being broadly discussed in the medical community. How aware of this are you?	13	29	67	63	14
To what extent do you feel you have an understanding of the technologies which	11	45	80	43	7

underpin 'Artificial Intelligence' and 'Deep Learning'?					
**Attitudes and feelings towards AI**					
I perceive Artificial Intelligence in Medicine as a partner rather than as a competitor.	2	8	42	61	73
Artificial intelligence will revolutionize Medicine/Dentistry in general.	0	4	13	73	96
The non-interventional physician will be replaced in the foreseeable future.	29	45	66	40	6
In the foreseeable future, all physicians will be replaced.	83	52	34	12	5
These developments frighten me.	38	58	43	35	12
These developments make Medicine in general more exciting to me.	6	8	27	102	43
Artificial intelligence will never make the human physician expendable.	6	27	50	48	55
Artificial intelligence will improve Medicine in general.	0	3	9	75	99
Artificial intelligence should be part of medical/dental training.	3	4	13	81	85

UNITED KINGDOM (n=331)					
**Understanding on AI basic principles**					
Artificial intelligence is an umbrella term encompassing many technologies (e.g., 'Machine Learning'). Do you have a basic understanding of these technologies?	18	100	125	61	27
Currently, AI has many applications in Medicine (e.g., AI-assisted robotic surgery). How familiar are you with these applications?	44	96	126	48	17
Many applications we use in daily life already use AI (e.g. speech-/text-recognition, email spam-filters). How familiar are you with these applications?	9	37	115	116	54
**AI as a topic in Medicine and Dentistry**					
AI in medical research is rapidly evolving. Healthcare AI projects attracted more investment than any AI projects in any other field globally. How aware are you?	39	93	117	69	13
‘Artificial Intelligence’ and ‘Deep Learning’ are currently being broadly discussed in the medical community. How aware of this are you?	34	93	114	70	20
To what extent do you feel you have an understanding of the technologies which	80	103	103	37	8

underpin 'Artificial Intelligence' and 'Deep Learning'?					
**Attitudes and feelings towards AI**					
I perceive Artificial Intelligence in Medicine as a partner rather than as a competitor.	6	30	64	114	117
Artificial intelligence will revolutionize Medicine/Dentistry in general.	0	13	27	143	148
The non-interventional physician will be replaced in the foreseeable future.	53	96	83	79	20
In the foreseeable future, all physicians will be replaced.	187	76	42	22	4
These developments frighten me.	79	101	62	71	18
These developments make Medicine in general more exciting to me.	11	27	75	128	90
Artificial intelligence will never make the human physician expendable.	12	45	79	104	91
Artificial intelligence will improve Medicine in general.	2	7	31	145	146
Artificial intelligence should be part of medical/dental training.	4	14	49	144	120

Only countries with more than 50 respondents are presented. Data is presented as counts.

AI, artificial intelligence.
